# Development and validation of radiomics models for the prediction of diagnosis of classic trigeminal neuralgia

**DOI:** 10.3389/fnins.2023.1188590

**Published:** 2023-10-09

**Authors:** Fuxu Wang, Anbang Ma, Zeyu Wu, Mingchen Xie, Peng Lun, Peng Sun

**Affiliations:** ^1^Department of Neurosurgery, Affiliated Hospital of Qingdao University, Qingdao, China; ^2^Shanghai Xunshi Technology Co., Ltd., Shanghai, China

**Keywords:** trigeminal neuralgia, MRI, radiology, machine learning, diagnosis

## Abstract

The study aims to develop a magnetic resonance imaging (MRI)-based radiomics model for the diagnosis of classic trigeminal neuralgia (cTN). This study involved 350 patients with cTN and 100 control participants. MRI data were collected retrospectively for all the enrolled subjects. The symptomatic side trigeminal nerve regions of patients and both sides of the trigeminal nerve regions of control participants were manually labeled on MRI images. Radiomics features of the areas labeled were extracted. Principle component analysis (PCA) and least absolute shrinkage and selection operator (LASSO) regression were utilized as the preliminary feature reduction methods to decrease the high dimensionality of radiomics features. Machine learning methods were established, including LASSO logistic regression, support vector machine (SVM), and Adaboost methods, evaluating each model’s diagnostic abilities using 10-fold cross-validation. All the models showed excellent diagnostic ability in predicting trigeminal neuralgia. A prospective study was conducted, 20 cTN patients and 20 control subjects were enrolled to validate the clinical utility of all models. Results showed that the radiomics models based on MRI can predict trigeminal neuralgia with high accuracy, which could be used as a diagnostic tool for this disorder.

## 1. Introduction

Trigeminal neuralgia (TN) is characterized by unilateral brief shock-like paroxysmal pain in one or more branches of the trigeminal nerve of the suffering patient (Maarbjerg et al., 2017). Trigeminal Neurovascular Compression is considered the cause of most TN by the International Headache Society (IHS). TN is diagnosed based on three main clinical symptoms: pain limited to one or more areas of the trigeminal nerve distribution; sudden and severe pain of very short duration, described as “shock” or “electrical conductivity”; and pain can be caused by innocuous stimulation of the trigeminal nerve distribution area in the face or mouth (Cruccu et al., 2020). TN is classified into three types according to their etiology: classic, secondary, and idiopathic. The most common classic trigeminal neuralgia (cTN) is caused by intracranial vascular compression of the trigeminal nerve root. Secondary TN accounts for approximately 15% of all cases and can be attributed to a neurological disease that can be clearly diagnosed, such as multiple sclerosis or a tumor in the pontocerebellar horn region compressing the trigeminal nerve. Idiopathic trigeminal neuralgia (ITN) is a neuropathic pain along the distribution of the trigeminal nerve with unknown pathogenesis and a part of ITN may be caused by NVC (ITN-NVC) (Ge et al., 2022).

Despite a known etiology of cTN and clear clinical symptoms, cTN is sometimes misdiagnosed due to its low incidence (Slettebo, 2021). Cranial nerve magnetic resonance imaging (MRI) is commonly used to exclude secondary TN and to aid in the diagnosis of cTN because it can show the contact between the trigeminal nerve and blood vessels. However, it has some limitations. However, many studies have shown that brain MRI cannot always accurately detect neurovascular contact (NVC) on the symptomatic side. In other words, the presence of neurological symptoms (negative likelihood ratio = 0.5) is not necessarily ruled out by the lack of MRI evidence of NVC (Antonini et al., 2014). This study showed poor performance of this diagnostic test for determining NVC’s presence, location, and type.

By enhancement of vascular inflow, the three-dimensional time-of-flight magnetic resonance angiography sequence technology (3D-TOF-MRA) brings out the high signal intensity of arteries and the average signal intensity of brain parenchyma and trigeminal nerve and the low signal intensity of cerebrospinal fluid (CSF). This can lead to better comparison and relationship assessment between arteries and nerves. However, its primary disadvantage is that as a result of the enhanced inflow effect, slow blood flow vessels are not displayed, which significantly reduces the diagnostic efficacy of MRI in patients with cTN having neurovascular compression (Zeng et al., 2021). Patel et al. analyzed the preoperative MRI scans of 92 TN patients prior to surgery. In nine patients, vascular compression was not detected by MRI findings but was found during surgery. It is clear thus that not all neurovascular compression is reflected on MRI (Lorenzoni et al., 2012). Consequently, although MRI is necessary for ruling out symptomatic TN, it cannot be utilized to diagnose cTN.

The term radiomics has been attracting much attention during the past several years. It converts medical images into high-dimensional recoverable data through high-throughput extraction of quantitative features and subsequent data analysis to provide decision support (Huang et al., 2016). More specifically, radiomics analysis is a process used to extract quantitative features from medical images using advanced feature extraction procedures based on machine learning algorithms, such as the least absolute shrinkage and selection operator (LASSO) or logistic regression methods. These methods are now used to build disease detection, classification models, prognosis prediction, and therapeutic response evaluation. Moreover, these methods have been widely used in assessing tumor stages, diagnosis, and metastasis.

Different MRI scan sequences have been evaluated to diagnose and predict the prognosis of cranial nerve disorders. Three-dimensional time-of-flight (3D-TOF) and three-dimensional constructive interaction in steady state (3D-CISS) sequences were studied by Jia et al. and analyzed for 95 patients with facial spasms. They found that 3D-TOF and 3D-CISS imaging had a 98.95% positive rate and a 100% overall accuracy in depicting the relationship between the facial nerve and surrounding vessels (Jia et al., 2016). Despite the radiomics model and machine learning techniques showing promising results in diagnosing cranial nerve-related disorders, there are few relevant studies establishing them as a full-fledged diagnosis model.

In this study, the MRI images of 350 cTN patients and 100 control subjects were collected retrospectively. Based on the dataset, the trigeminal nerve regions of interest (ROIs) were manually labeled and radiomics features were extracted. Radiomics models using machine learning methods were established and the diagnostic performance of all the models was evaluated. Principle component analysis (PCA) and LASSO regression methods were employed to perform dimension reduction of high dimensional radiomics features with exploring the impact on performance when building the radiomics models. A prospective study was conducted to validate the clinical utility of the established radiomics models with 20 cTN patients and 20 control subjects enrolled. The research can aid the current literature on imaging diagnosis of cTN to help patients and clinicians make informed decisions before any intervention.

## 2. Materials and methods

### 2.1. Participants

The present study protocol received ethical approval from the Ethics Committee of the Affiliated Hospital of Qingdao University. All participants agreed to provide their written informed consent for participation in the study.

The patient inclusion criteria were as follows: (1) patients showing typical symptoms of TN; (2) patients diagnosed with cTN via microvascular decompression of the trigeminal nerve; and (3) patients with complete imaging data before surgery.

For control participants, the inclusion criteria were as follows: (1) patients coming for treatment with benign paroxysmal positional vertigo (BPPV); (2) individuals without a history of facial pain; (3) cranial nerve MRI was performed and the original image completely preserved; and (4) individuals without TN disorders as determined by experienced radiologists using cranial nerve MRI images.

The exclusion criteria for the study were as follows: (1) typical TN symptoms, but not confirmed by surgery; (2) diagnosed as secondary TN; (3) incomplete preservation of image and clinical data. The exclusion criteria for the control group were as follows: (1) patients who had undergone trigeminal nerve surgery and (2) patients with incomplete preservation of the general data.

### 2.2. Clinical data evaluation and grouping

Retrospective analyses were conducted by using the clinical data of all patients, including the age, gender, symptoms, and duration of disease. The enrolled patients all met the diagnostic criteria in accordance with the International Classification of Headache Disorders (3rd edition; ICHD-3) (Headache Classification Committee of the International Headache Society [IHS], 2013). The diagnostic criteria for cTN from The International Classification of Headache Disorders comprise the following:

A. At least 3 attacks of unilateral facial pain fulfilling criteria B and C.
B. Occurring in one or more divisions of the trigeminal nerve, with no radiation beyond the trigeminal distribution.
C. Pain presenting with at least 3 of the following 4 characteristics:
  1. recurrence in paroxysmal attacks lasting from a fraction of a second to 2 min;
  2. severe intensity;
  3. electric shock-like, shooting, stabbing, or sharp quality;
  4. precipitated by innocuous stimuli to the affected side of the face.
D. No clinically evident neurological deficit.
E. Not better accounted for based on another ICHD-3 diagnosis.

All patients selected in this study received trigeminal microvascular decompression. They showed symptoms of TN before the operation and were confirmed to be cTN during the operation. The pain symptoms of 315 patients disappeared at the time of the operation, while the pain symptoms of 2 patients did not change after the operation.

In the prospective study, 20 cTN patients and 20 control subjects were enrolled. For all the cTN patients, the pain symptoms disappeared at the time of the operation.

### 2.3. MRI acquisition

Magnetic resonance images of all subjects were collected retrospectively. MRI images performed within 48 h prior to microvascular decompression were collected for TN patients. For control subjects, their previous MRI images at the first outpatient visit were collected. The subjects who met this criterion (which included 350 cTN patients and 100 control participants) were selected for routine MRI scans to obtain their nerve magnetic resonance sequence and vascular magnetic resonance sequence using the GE (General Electric Healthcare, Milwaukee, WI, USA) Signa 1.5T/3.0T scanners and Siemens (Siemense Healthcare, Henkestr, Erlangen, Germany) Aera 1.5T/3.0T scanner. The nerve magnetic resonance sequence with the GE Signa scanners was the Fiesta-C sequence. The nerve magnetic resonance sequence using the Siemense Aera scanners was the t2-spc-tra-iso sequence. The vascular magnetic resonance sequence with the GE Signa scanners was 3D-TOF. The vascular magnetic resonance sequence with the Siemense Aera scanners was TOF-multi-slab.

For the retrospective study, in the control group, there were 25 subjects with 1.5-T GE scanners, 30 with 3.0-T GE scanners, 20 with 1.5-T Siemens scanners, and 25 with 3.0-T Siemense scanners. In the cTN group, with GE scanners, 81 MRI images were obtained with a 1.5-T field strength and 104 with a 3.0-T field strength, while, with the Siemense scanners, 75 images were obtained with a 1.5-T field strength, and 90 with a 3.0-T field strength.

For the prospective validation study, in the control group, there were 4 subjects with 1.5-T GE scanners, 8 with 3.0-T GE scanners, 3 with 1.5-T Siemens scanners, and 5 with 3.0-T Siemense scanners. In the cTN group, with GE scanners, 5 MRI images were obtained with a 1.5-T field strength and 8 with a 3.0-T field strength, while, with the Siemense scanners, 2 images were obtained with a 1.5-T field strength, and 5 with a 3.0-T field strength.

The parameters of the Fiesta-C sequence were as follows: TR-3.5 ms, TE-1.5 ms, FA-60°, FOV-22–24 cm, and 0.7-mm slice thickness. The parameters of a 3D-TOF sequence included the following: TR-23 ms, TE-3.6M ms, FA-15°, FOV-22–24 cm, and 0.7-mm slice thickness.

The parameters of the t2-spc-tra-iso sequence were as follows: TR-1,000 ms, TE-266 ms, FA-150°, FOV-20–22 cm, and 0.6–0.8-mm slice thickness. The parameters of the TOF-multi-slab sequence scan were as follows: TR-9 ms, TE-2.39 ms, FA-25°, FOV-20–22 cm, and 0.6–0.8-mm slice thickness.

### 2.4. Neuroimage processing

#### 2.4.1. Manual segmentation of the region of interest

**FIGURE 1:**
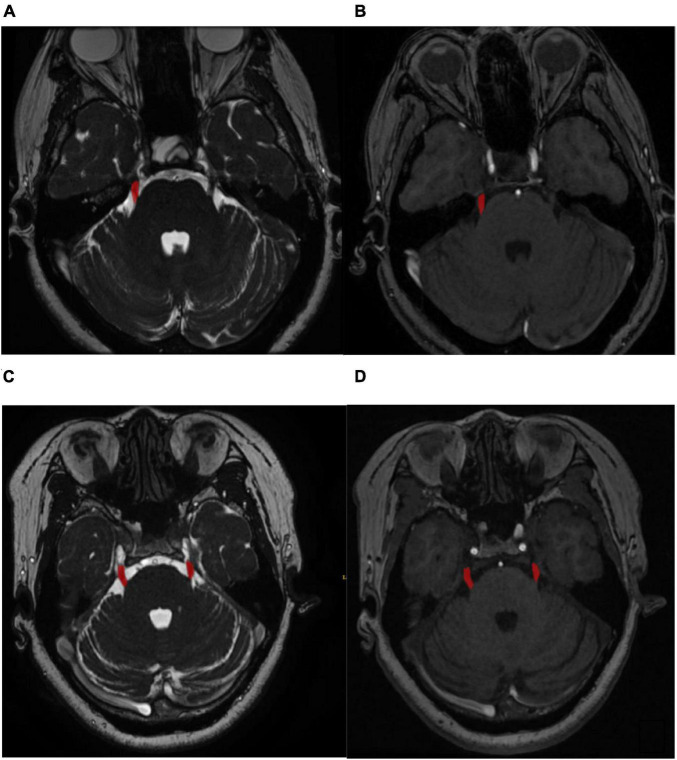
**(A)** Labeled symptomatic side regions of nerve magnetic resonance sequence. **(B)** Labeled symptomatic side regions of vascular magnetic resonance sequence. **(C)** Labeled both the left and right sides regions of nerve magnetic resonance sequence. **(D)** Labeled both the left and right sides regions of vascular.

For the retrospective study, the nerve magnetic resonance sequence and the vascular magnetic resonance sequencing images were manually labeled with ITK-SNAP^1^ by three experienced radiologists. The labeled masks were finally examined by an experienced neurosurgeon. The labeled ROI in the nerve magnetic resonance sequence and the vascular magnetic resonance sequence included the symptomatic side nerve ROI for the cTN group and both the left and right sides nerve ROI for the control group (Figure 1, for example, images). Therefore, we achieved 350 ROIs for cTN and 200 ROIs for the control group to establish the retrospective training dataset.

For the prospective study, the MRI images were still manually labeled with ITK-SNAP software by two experienced radiologists. The labeled masks were finally checked by another experienced neurosurgeon. The symptomatic side nerve ROIs of the cTN patients and random side nerve ROIs of the control subjects from both nerve and vascular magnetic resonance sequences were labeled to form a prospective validation dataset.

#### 2.4.2. Radiomics feature extraction and analysis

**FIGURE 2:**
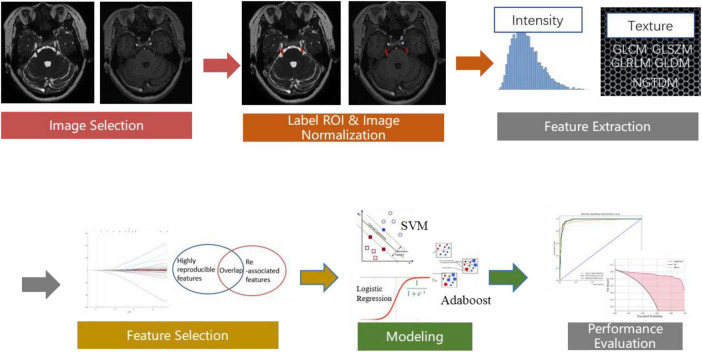
The workflow chart depicting the process of radiomics feature extraction and analysis.

The workflow of radiomics feature extraction and prediction modeling is depicted in Figure 2. In the feature extraction procedure, all Digital Imaging and Communications in Medicine (DICOM) formatted images were resampled with the same pixel spacing (0.5 × 0.5 × 0.5 mm). The quantile normalization method was utilized to realize pixel-intensity normalization for both nerve and vascular MRI sequences. The procedures followed for intensity normalization were as follows:


min_value=q⁢u⁢a⁢n⁢t⁢i⁢l⁢e⁢(i⁢m⁢a⁢g⁢e, 0.001)

max⁢_⁢v⁢a⁢l⁢u⁢e=q⁢u⁢a⁢n⁢t⁢i⁢l⁢e⁢(i⁢m⁢a⁢g⁢e,0.999)

$$i\,f\,P\,i\,x\,e\,l_{s\,r\,c}>\max\,\_\,\mathrm{value}:P\,i\,x\,e\,l_{s\,r\,c}=\text{max\_value}$$

$$i\,f\,P\,i\,x\,e\,l_{s\,r\,c}<\min\,\_\,\mathrm{value}:P\,i\,x\,e\,l_{s\,r\,c}=\text{min\_value}$$

$$Pixel_{d\,s\,t}=round(\left(Pixel_{s\,r\,c}-\text{min}\_value\right)/$$

(max_value-min_value)*512)

Where the image is the input-source MRI image, the quantile function was used to get min_value (corresponding to 0.001 probs), and max_value (corresponding to 0.999 probs). Then, for each pixel of the input source MRI images, min_value, and max_value were used to normalize the pixel intensity from 0 to 512.

Pyradiomics toolkit^2^ was used to extract 200 radiomics features for each ROI, including 14 shape and size features related to the 3D size and shape of the ROI, 36 first-order features based on the distribution of voxel intensities calculated from nerve and vascular MRI sequences, 150 texture-based features of both nerve and vascular MRI sequences that were calculated from the gray-level co-occurrence matrix (GLCM), gray-level run-length matrix (GLRLM), gray-level size-zone matrix (GLSZM), gray-level dependence matrix (GLDM), and neighborhood gray-tone difference matrix (NGTDM). The abovementioned features have been confirmed to potentially reflect changes in the image structure. In the feature selection and feature dimension reduction procedure, Pearson’s correlational analysis and Spearman correlation coefficient analysis were employed to eliminate poorly correlated and repeated radiomics features. Finally, 104 robust predictive radiomics features were selected to build the imaging diagnosis model of cTN.

For the prospective validation dataset, the image normalization process and radiomics feature extraction method were consistent with the training dataset. However, feature selection and feature dimension reduction procedures were not employed for the prospective validation. The 104 corresponding predictive radiomics features were manually remained to validate the performance of the imaging diagnosis model of cTN, instead.

### 2.5. cTN imaging diagnosis model

For a large set of high-dimensional potential predictors, there are many conventional methods applied for variable selection, such as stepwise selection. However, they have the disadvantage of overfitting (McNeish, 2015). LASSO (Tibshirani, 1996) is a variable selection method that can handle a large dataset with high-dimensional features, extracting the variables most associated with the disease (Algamal and Lee, 2015; Chong et al., 2021; Ouyang et al., 2022). The logistic regression with the adaptive LASSO is a frequently used method to establish the disease prediction or diagnostic model (Algamal and Lee, 2015), especially in radiomics-related research (She et al., 2018; Ji et al., 2019; Wu et al., 2019). Support vector machine (SVM) and Adaboost methods were also employed to build the cTN imaging diagnosis model. However, the high-dimensional data are composed of a large number of features while some of them are still redundant and increase the size and complexity of the feature space (Salimi et al., 2018). The high dimensionality could lead to the curse of dimensionality which will decrease the performance of the SVM and Adaboost methods (Gheyas and Smith, 2010). High accuracy does not imply that the features used are better than random (Ho et al., 2020). Thus, we applied principal component analysis (PCA) and LASSO regression as the preliminary feature reduction methods and explored the impact on the performance of the SVM and Adaboost methods. There were seven modeling methods in total (LASSO logistic regression, SVM, Adaboost, PCA-SVM, PCA-Adaboost, LASSO-SVM, and LASSO-Adaboost). Then, 10-fold cross-validation was conducted to test the performance of all models after the calculation of the ROC curve and AUC value. Confusion matrix of all models were utilized to calculate the accuracy, sensitivity, specificity, and F1-score to evaluate the performance. The decision curve analysis (DCA) was employed to quantify the clinical utility values of the models by calculating the greatest net benefit.

### 2.6. Statistical analyses

Statistical analyses were performed by using SPSS software (version 26.0). The gender distribution and MRI sequences-type distribution of the two groups were compared by Chi-squared test. Student’s *t*-tests or Mann–Whitney U-tests were performed for continuous variables of the two groups. A two-sided *P* < 0.05 was considered to indicate statistical significance. ROC curve analysis was performed to evaluate the diagnostic performances of the models. The DCA was applied to quantify the clinical utility value.

## 3. Results

### 3.1. Patient characteristics

For both the retrospective training dataset and the prospective validation dataset, cTN patients and control participants were similar in terms of gender, age distribution, and use of MRI scanners. The cTN imaging diagnosis model was developed using the symptomatic side nerve ROI of cTN patients and the left and right sides nerve ROI of control participants from the retrospective training dataset. The cTN imaging diagnosis model was externally validated using the symptomatic side nerve ROI of cTN patients and random side nerve ROI of control participants from the prospective validation dataset.

The patient characteristics of the retrospective training dataset and prospective validation dataset were shown in Tables 1, 2. There was no statistical correlation between the two groups as for as the gender (*P* = 0.162) and age (*P* = 0.076) of patients were concerned for both datasets. As can be seen from the results shown in Tables 1, 2, which detail baseline data for all participants, no data are statistically significant (*P* = 0.05).

**TABLE 1:** Clinical characteristics of the cTN and control group participants of retrospective training dataset.

	Total	cTN patients (350)	Healthy participants (100)	*P*
Age (mean ± SD)	56.65 ± 10.65	57.27 ± 9.05	54.48 ± 14.80	0.076
Sex, no. (%)				0.162
Male	189 (42.0)	141 (40.2)	48 (48)	
Female	261 (58)	209 (59.7)	52 (52)	
Image sequence (nerve) (%)				0.705
Fiesta-C*	240 (53.3)	185 (52.9)	55 (55)	
t2_spc_tra_iso*	210 (46.7)	165 (47.1)	45 (45)	
Image sequence (vessel) (%)				0.705
3D-TOF*	240 (53.3)	185 (52.9)	55 (55)	
TOF_3D_multi-slab*	210 (46.7)	165 (47.1)	45 (45)	
MRI type (%)				0.976
1.5T GE scanners	106 (23.6)	81 (23.1)	25 (25)	
3.0T GE scanners	134 (29.8)	104 (29.7)	30 (30)	
1.5T Siemense scanners	95 (21.1)	75 (21.4)	20 (20)	
3.0T Siemense scanners	115 (25.6)	90 (25.7)	25 (25)	

*The Fiesta-C and 3D-TOF sequences correspond to GE’s magnetic resonance. The t2_spc_tra_iso and TOF_3D_multi-slab sequences correspond to Siemens’ magnetic resonance.

**TABLE 2:** Clinical characteristics of the cTN and group participants of prospective validation dataset.

	Total	cTN patients (20)	Control participants (20)	*P*
Age (mean ± SD)	57.4 ± 12.95	59.03 ± 12.81	55.78 ± 13.06	0.135
Sex, no. (%)				0.525
Male	18 (45.0)	10 (50.0)	8 (40.0)	
Female	22 (55.0)	10 (50.0)	12 (60.0)	
Image sequence (nerve) (%)				0.744
Fiesta-C*	25 (62.5)	13 (65.0)	12 (60.0)	
t2_spc_tra_iso*	15 (37.5)	7 (35.0)	8 (40.0)	
Image sequence (vessel) (%)				0.744
3D-TOF*	25 (62.5)	13 (65.0)	12 (60.0)	
TOF_3D_multi-slab*	15 (37.5)	7 (35.0)	8 (40.0)	
MRI type (%)				0.989
1.5T GE scanners	9 (22.5)	5 (25.0)	4 (20.0)	
3.0T GE scanners	16 (40.0)	8 (40.0)	8 (40.0)	
1.5T Siemense scanners	5 (12.5)	2 (10.0)	3 (15.0)	
3.0T Siemense scanners	10 (25.0)	5 (25.0)	5 (25.0)	

*The Fiesta-C and 3D-TOF sequences correspond to GE’s magnetic resonance. The t2_spc_tra_iso and TOF_3D_multi-slab sequences correspond to Siemens’ magnetic resonance.

### 3.2. Diagnostic efficacy of radiomics-based models for diagnosis of cTN

In the training procedure, a total of 200 radiomics features were obtained from nerve and vascular images. From the original set of 200 radiomics features, 104 were selected by eliminating those with poor response-correlations and that were repetitions. The imaging-based cTN diagnosis model was developed using LASSO logistic regression, SVM, Adaboost, PCA-SVM, PCA-Adaboost, LASSO-SVM, and LASSO-Adaboost.

**FIGURE 3:**
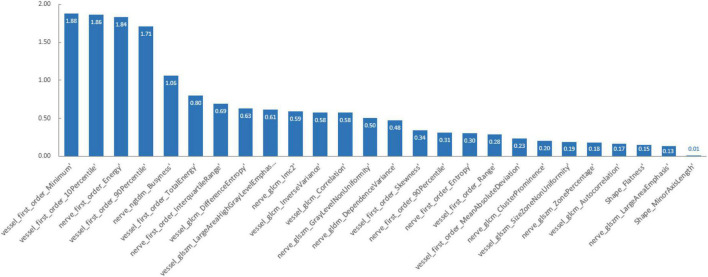
The weights of radiomics features.

Principal component analysis and LASSO regression as the preliminary feature reduction methods were employed to decrease the dimension of radiomics features while exploring the impact on the performance of the SVM and Adaboost methods. For PCA analysis, 95% of the variance in the data was preserved and the selected number of components was 24. 26 radiomics features were selected and retained after LASSO regression with assigning weights to each feature. The feature weights were ranked from largest to smallest in Figure 3.

The PCA-SVM and PCA-Adaboost were built based on the PCA-dataset with a feature dimensionality of 24. The LASSO-SVM and LASSO-Adaboost were built based on the LASSO-dataset with a feature dimensionality of 26.

**FIGURE 4:**
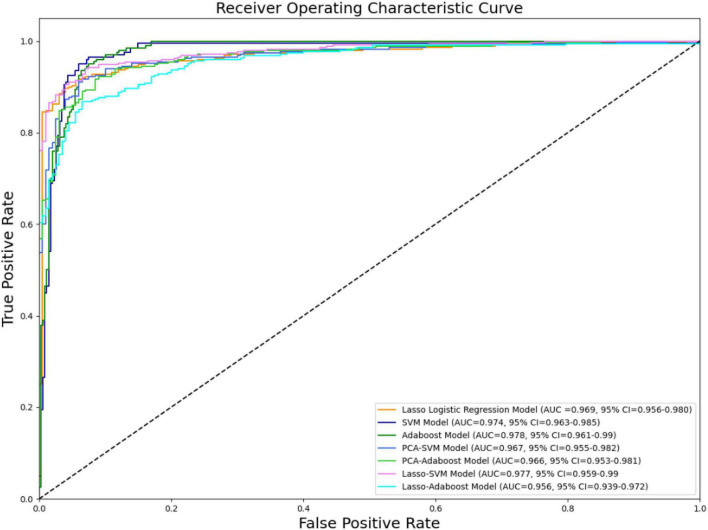
ROC curve of all models.

The effectiveness of the model’s performance was evaluated using a 10-fold cross-validation procedure. The accuracy, sensitivity, specificity, precision, F1-score, and AUC of all models are shown in Table 3. Moreover, the SVM model and Adaboost model outperformed the LASSO logistic regression model in the assessment (Figure 4). However, the preliminary feature reduction methods did not impact the performance of SVM and Adaboost significantly.

**TABLE 3:** The AUC, accuracy, sensitivity, specificity, precision, and F1-score of all models.

Model	AUC (95% CI)	Accuracy	Sensitivity	Specificity	Precision	F1-score
LASSO logistic regression	0.969 (0.956–0.980)	0.9182	0.9257	0.905	0.9446	0.9351
SVM	0.974 (0.963–0.985)	0.94	0.9429	0.935	0.9621	0.9524
Adaboost	0.978 (0.961–0.99)	0.9363	0.9314	0.945	0.9674	0.9491
PCA-SVM	0.967 (0.955–0.982)	0.9182	0.943	0.875	0.9296	0.9362
PCA-Adaboost	0.966 (0.953–0.981)	0.9091	0.92	0.89	0.936	0.928
LASSO-SVM	0.977 (0.959–0.99)	0.9273	0.9229	0.935	0.9613	0.9417
LASSO-Adaboost	0.956 (0.939–0.972)	0.8854	0.8686	0.915	0.947	0.9061

**FIGURE 5:**
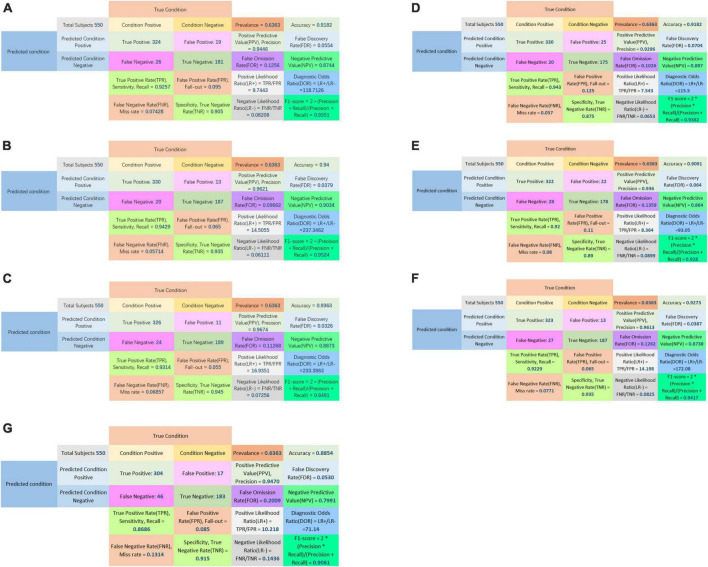
Confusion matrix of LASSO logistic regression model **(A)**, SVM model **(B)**, Adaboost model **(C)**, PCA-SVM model **(D)**, PCA-Adaboost model **(E)**, LASSO-SVM model **(F)**, and LASSO-Adaboost model **(G)**.

The confusion matrices of all classifiers were plotted in Figure 5.

**FIGURE 6:**
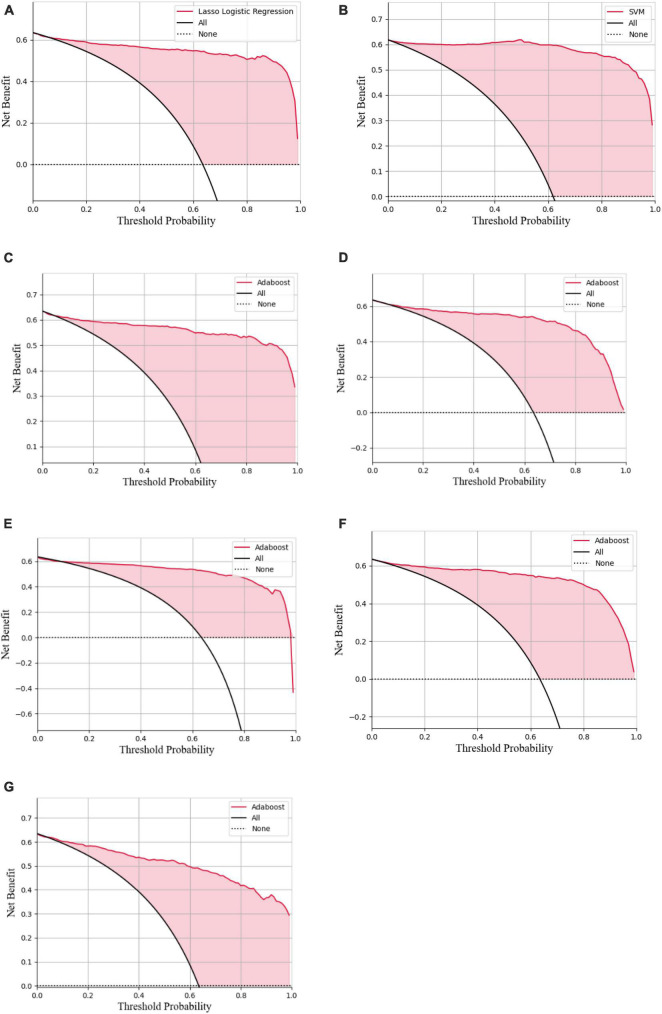
Decision curve analysis of the LASSO logistic regression model **(A)**, SVM model **(B)**, Adaboost model **(C)**, PCA-SVM model **(D)**, PCA-Adaboost model **(E)**, LASSO-SVM model **(F)**, and LASSO-Adaboost model **(G)**.

In the decision curve plot, all models showed high values of clinical utility (Figure 6).

### 3.3. External validation of radiomics-based models for diagnosis of cTN

**FIGURE 7:**
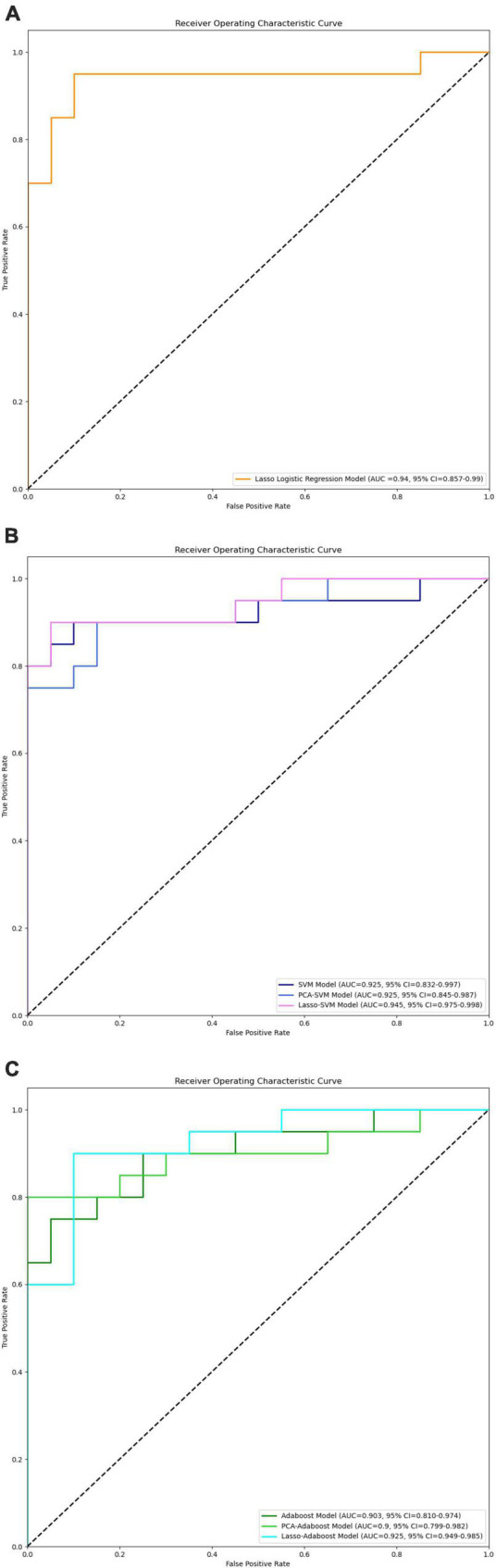
ROC curves of LASSO logistic regression **(A)**, SVM, PCA-SVM, and LASSO-SVM **(B)**, Adaboost, PCA-Adaboost, and LASSO-Adaboost **(C)** models based on prospective validation dataset.

**FIGURE 8:**
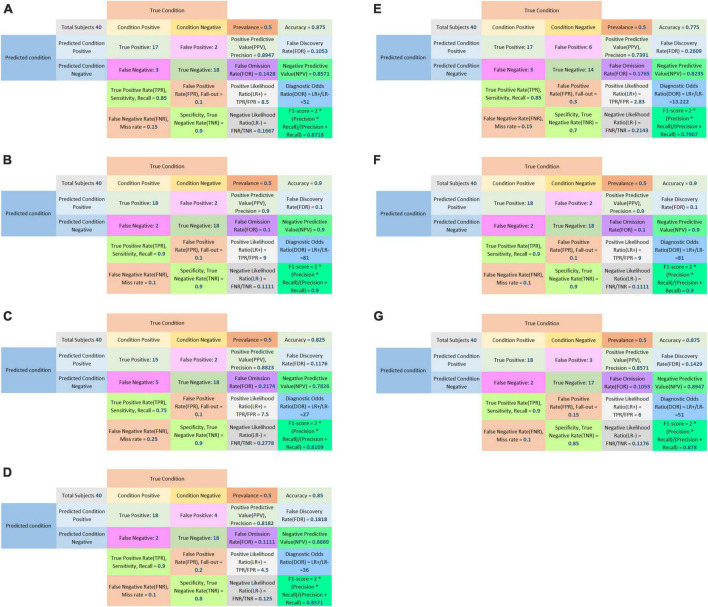
Confusion matrix of LASSO logistic regression model **(A)**, SVM model **(B)**, Adaboost model **(C)**, PCA-SVM model **(D)**, PCA-Adaboost model **(E)**, LASSO-SVM model **(F)**, and LASSO-Adaboost model **(G)** based on prospective validation dataset.

**FIGURE 9:**
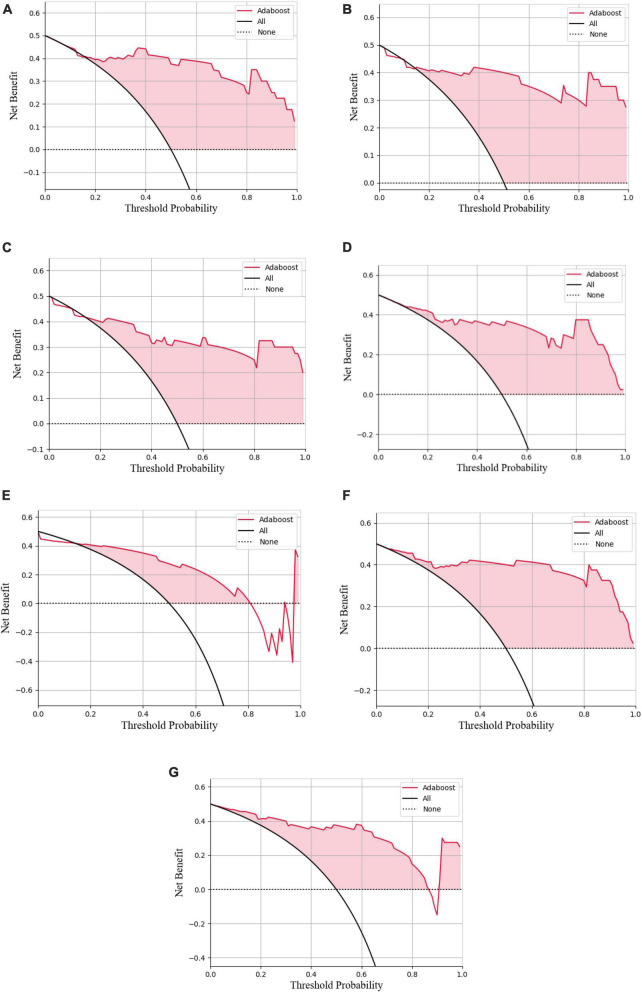
Decision curve analysis of the LASSO logistic regression model **(A)**, SVM model **(B)**, Adaboost model **(C)**, PCA-SVM model **(D)**, PCA-Adaboost model **(E)**, LASSO-SVM model **(F)**, and LASSO-Adaboost model **(G)** based on prospective validation dataset.

In the external validation procedure, a total of 200 radiomics features were obtained from nerve and vascular images of the validation dataset. A total of 104 corresponding predictive radiomics features were manually remained to validate the performance of the models. The effectiveness of the model’s performance was evaluated using the scores of accuracies, sensitivity, specificity, precision, F1-score, and AUC. The scores are listed in Table 4. The diagnostic power of all models in predicting cTN is shown in Table 4 and Figures 7–9. The preliminary feature reduction methods did not impact the performance of SVM and Adaboost significantly in the external validation.

**TABLE 4:** The AUC, accuracy, sensitivity, specificity, precision, and F1-score of all models based on prospective validation dataset.

Model	AUC (95% CI)	Accuracy	Sensitivity	Specificity	Precision	F1-score
LASSO logistic regression	0.94 (0.857–0.99)	0.875	0.85	0.9	0.8947	0.8718
SVM	0.925 (0.832–0.997)	0.9	0.9	0.9	0.9	0.9
Adaboost	0.903 (0.810–0.974)	0.825	0.75	0.9	0.8823	0.8109
PCA-SVM	0.925 (0.845–0.987)	0.85	0.9	0.8	0.8182	0.8571
PCA-Adaboost	0.9 (0.799–0.982)	0.775	0.85	0.7	0.7391	0.7907
LASSO-SVM	0.945 (0.975–0.998)	0.9	0.9	0.9	0.9	0.9
LASSO-Adaboost	0.925 (0.949–0.985)	0.875	0.9	0.85	0.8571	0.878

The external validation confusion matrices of all classifiers were plotted in Figure 8. In the decision curve plot, all models showed high values of clinical utility (Figure 9) with the external validation dataset.

## 4. Discussion

This research confirms that trigeminal nerve MRI radiomics feature extraction and the resulting radiomics model strongly correlate with cTN diagnosis. In this study, we retrospectively gathered trigeminal nerve imaging data from 350 patients with cTN and 100 control participants, extracted the imaging features, and built three advanced binary classification models: the LASSO logistic regression, SVM, and Adaboost models. The accuracies were 91.78% (AUC = 0.969, 95% CI = 0.956–0.980), 94% (AUC = 0.974, 95% CI = 0.963–0.985), and 93.63% (AUC = 0.978, 95% CI = 0.961–0.99), respectively. PCA and LASSO regression were employed as the preliminary feature reduction methods to reduce the dimension of radiomics features to 24 and 26, respectively. The SVM and Adaboost models were established based on the feature reduction datasets. The accuracies of the models were 91.82% (AUC = 0.967, 95% CI = 0.955–0.982), 90.91% (AUC = 0.966, 95% CI = 0.953–0.981), 92.73% (AUC = 0.977, 95% CI = 0.959–0.99), and 88.54% (AUC = 0.956, 95% CI = 0.939–0.972) for PCA-SVM, PCA-Adaboost, LASSO-SVM, and LASSO-Adaboost, respectively. Our finding demonstrates the efficacy of the radiomics-based predictive models that can accurately diagnose cTN.

To validate the clinical utility, a prospective study was conducted with 20 cTN patients and 20 control participants enrolled. Image radiomics features were extracted and applied to validate the cTN predictive models. The accuracies were 87.5% (AUC = 0.94, 95% CI = 0.857–0.99), 90.0% (AUC = 0.925, 95% CI = 0.832–0.997), 82.5% (AUC = 0.903, 95% CI = 0.810–0.974), 85.0% (AUC = 0.925, 95% CI = 0.845–0.987), 77.5% (AUC = 0.9, 95% CI = 0.799–0.982), 90.0% (AUC = 0.945, 95% CI = 0.975–0.998), and 87.5% (AUC = 0.925, 95% CI = 0.949–0.985) for LASSO logistic regression, SVM, Adaboost, PCA-SVM, PCA-Adaboost, LASSO-SVM, and LASSO-Adaboost, respectively. The external validation proved the accuracy and clinical value of the radiomics-based predictive models. However, we found that the accuracy of the external validation set was lower than that of the training set, which may be caused by the fact that the model constructed in this study was slightly biased compared to the actual situation due to the small number of subjects.

Facial TN is a form of chronic neuropathic pain. The International Association for the Study of Pain defines typical idiopathic TN as “sudden, usually unilateral, severe, transient, stabbing and recurrent pain in one or more branches of the fifth cranial nerve.” Only 12.6 new cases per 100,000 persons per year indicate a low incidence rate (Koopman et al., 2009). It has been reported that nerve demyelination caused by NVC produces ectopic impulses traveling as brain waves is the basis for triggering cTN attacks (Cao et al., 2021). Neurovascular compression has been proven to be the main cause of cTN (Cruccu et al., 2016). Although the clinical symptoms of cTN are obvious and the diagnosis of cTN is mainly based on clinical symptoms, it is subjective in nature and has more differential diagnoses. These include: glossopharyngeal neuralgia, painful posttraumatic trigeminal neuropathy, persistent idiopathic facial pain, painful trigeminal neuropathy attributed to acute herpes zoster, short-lasting unilateral neuralgiform headache attacks with autonomic symptoms (SUNA), short-lasting unilateral neuralgiform headache attacks with conjunctival injection and tearing (SUNCT) or paroxysmal hemicrania, cluster headache, primary stabbing headache, cracked tooth, caries or pulpitis (Maarbjerg et al., 2017). So a cTN diagnosis may be misdiagnosed due to performance changes that are not easy to diagnose.

Cranial nerve MRI is currently the gold standard for determining the degree of neurovascular compression, but it has drawbacks. The studies conducted by Hitchon et al. (2019) demonstrated that the specificity of MRI in determining the extent of neurovascular compression is only 50%. Although NVC is the most common cause of TN, patients may still show negative images, ruling out trigeminal microvascular decompression and the possibility of a complete recovery. Although Maarbjerg et al. (2015) had shown that severe NVC was more common on the symptomatic side, they also found that NVC was common on both sides of symptomatic and asymptomatic sides of healthy people and most patients. The final diagnosis in traditional imaging is subjective and based on the opinions of the imaging experts who perform the procedure. The next course of treatment for a patient with cTN may be affected by the clinician’s diagnosis, which may differ from those of other clinicians who have examined the patient. Therefore, developing a model based on feature information (radiomics) through imaging to diagnose cTN is crucial (Barker et al., 1996).

The majority of previous research has concentrated on the use of imaging to assess which vessels are at fault. To better demonstrate the position of compression in the facial nerve root exit zone (fREZ), Teton et al. (2019) performed a 3D reconstruction of MR and MRA images from 35 patients. Similarly, Satoh et al. (2007) also conducted a 3D reconstruction of nerves and blood vessels in patients with TN, demonstrating the relationship between the trigeminal nerve and blood vessels in some representative cases. Zhao et al. (2022) analyzed the MRI data from 67 patients with unilateral TN to identify such vessels and established a prediction model based on this data. Hughes reported a case study in which the superior cerebellar artery (SCA) was in contact with the trigeminal nerve at the position close to the trigeminal foramen. However, the presence of the protective peripheral myelin sheath around the nerve meant that it could resist the influence of vascular compression on the nerve. Thus, it was still a remote possibility making TN less likely to occur (Hughes et al., 2016). Hitchon et al. (2015) found that of 51 patients, 8 had MRI results that contradicted or otherwise did not match the vessels thought to be at fault after surgery. As such, the prediction diagnosis of cTN was not included in any prior research and instead only focused on its etiology.

Radiomics and machine learning studies at present mainly focus on the differential diagnosis, genotype, or survival rate of tumors (Xu et al., 2021). Using diffusion-weighted imaging (DWI) and 18F fluorodeoxyglucose positron emission tomography (18F FDG PET), Zhang et al. have developed an integrated radiomics model. To achieve a 98% (AUC = 0.98) accuracy rate in differentiating glioblastoma (GBM) from solitary brain metastases (SBM), he has built seven radiomics models and five non-radiomics techniques (Zhang et al., 2021). Shin et al. used radiomics to predict the prognosis of gastric cancer. The retrospective study used a training cohort (349 patients) and an external validation cohort (61 patients). Using 10-fold cross-validation and penalized Cox regression with the least absolute shrinkage and a selection operator, a radiomics model was developed. Results showed that its prediction efficiency was significantly higher than the clinical model (*P* < 0.01) (Shin et al., 2021). In general, current radiomics and machine learning research focus on predicting tumors’ differential diagnosis, genotype, or survival rate. There have been several radiomics studies on cTN. Mulford et al. used a convolutional U-net deep learning network to segment the trigeminal nerve from the pons to the ganglion. A radiomics approach was used to identify symptomatic trigeminal nerves from the MRIs of a group of TN patients, and the validation AUC of this model was 0.83, with sensitivities and specificities of 0.82 and 0.76, respectively (Mulford et al., 2022). Ge et al. (2022) explored risk factors for unilateral cTN or ITK-NCV with bilateral NVC using machine learning (ML), pointing out that textural features of trigeminal cisternal segments such as SALGLE, Coarseness, MAL, DV, Maximum, and Correlation, and the type of the offending vessel may be risk factors for cTN or ITK-NCV. Our study can provide an important addition to the existing literature.

This study proposes a new method for diagnosing cTN using an accurate predictive model based on MRI radiomics and machine learning tools. Radiomics can glean more detailed information than the conventional method of visually assessing images. Out of the 200 radiologic characteristics examined, 104 were found to be related to the diagnosis of cTN. Most of these features are not visible to the naked eye but are essential for a thorough assessment of a patient’s condition. We developed three advanced binary classification machine-learning algorithms and tested and verified their accuracy, performance, and clinical utility value. PCA and LASSO regression were employed as the preliminary feature reduction methods to reduce the dimension of radiomics features to 24 and 26, respectively. The SVM and Adaboost models were also established based on the feature reduction datasets. All the models demonstrated outstanding diagnostic ability, which means MRI radiomics is helpful in diagnosing cTN in clinical. The preliminary dimensional reduction methods did not impact the performance of SVM and Adaboost significantly. Even the AUC of the LASSO-SVM model is higher than that of the SVM model. The LASSO regression assigns weights to each feature when processing the step of feature selection. The different weights of features imply that certain features contribute more to the classification model than other features among the 104 radiomics features. By analyzing the weights of the remaining features, it could be found that first-order features extracted from vascular magnetic resonance images are the most distinctive ones. First-order statistics features describe the distribution of voxel intensities within the ROI regions. Intuitively, voxels with high intensity refer to cranial vessels in vascular magnetic resonance. Nevertheless, the small vessels cannot be identified by the naked eye due to low image resolution. The first-order statistics features are sensible to the changes in intensities, which indicates the possibility of small vessels. Thus, first-order statistics features might be the most significant features related to the diagnosis of cTN.

The conducted prospective study further validates the performance and clinical utility of all models. It can be seen that LASSO-SVM and LASSO-Adaboost demonstrated better performance than SVM and Adaboost. The high dimensionality led to the curse of dimensionality, which makes the SVM and Adaboost models unstable and less robust. Fewer features with higher weights assigned by LASSO regression will make the SVM and Adaboost models better generalization performance.

Although our study depicted ideal results, it still has some limitations. First, larger data sets are required to evaluate and adjust our model because the total number of samples for this radiomics study is very few. Finally, a larger, prospective, multicenter study on more patients is warranted to verify our findings.

## 5. Conclusion

In conclusion, this work points to the importance of the construction of the imaging omics model, and its essential role in diagnosing cranial nerve disorders. When clinical diagnosis is challenging, the radiomics model built by extracting features from MRI images might serve as a valuable adjunct due to its superior diagnostic efficiency compared to conventional diagnostic approaches for cTN.

## Data availability statement

The raw data supporting the conclusions of this article will be made available by the authors, without undue reservation.

## Ethics statement

The studies involving human participants were reviewed and approved by the Affiliated Hospital of Qingdao University. Written informed consent for participation was not required for this study in accordance with the national legislation and the institutional requirements.

## Author contributions

FW was responsible for the writing of the manuscript. AM was responsible for building machine learning models. ZW and MX were responsible for collecting imaging data. PL and PS were responsible for the revision of the manuscript and finalized the first draft. All authors contributed to the article and approved the submitted version.

## Footnotes

1
http://itksnap.org/

2
http://PyRadiomics.readthedocs.io/en/latest/
